# High-throughput screening of microbial strains in large-scale microfluidic droplets

**DOI:** 10.3389/fbioe.2023.1105277

**Published:** 2023-03-10

**Authors:** Zhidong Zhang, Qi Guo, Yuetong Wang, He Huang

**Affiliations:** ^1^ College of Biotechnology and Pharmaceutical Engineering, Nanjing Tech University, Nanjing, China; ^2^ School of Food Science and Pharmaceutical Engineering, Nanjing Normal University, Nanjing, China; ^3^ Institute of Applied Microbiology, Xinjiang Academy of Agricultural Sciences/ Xinjiang Laboratory of Special Environmental Microbiology, Urumqi, Xinjiang, China

**Keywords:** microfluidics, droplets, high-throughput screening, microbe, green biomanufacturing

## Abstract

The transformation of engineered microbial cells is a pivotal link in green biomanufacturing. Its distinctive research application involves genetic modification of microbial chassis to impart targeted traits and functions for effective synthesis of the desired products. Microfluidics, as an emerging complementary solution, focuses on controlling and manipulating fluid in channels at the microscopic scale. One of its subcategories is droplet-based microfluidics (DMF), which can generate discrete droplets using immiscible multiphase fluids at kHz frequencies. To date, droplet microfluidics has been successfully applied to a variety of microbes, including bacteria, yeast, and filamentous fungi, and the detection of massive metabolites of strain products, such as polypeptides, enzymes, and lipids, has been realized. In summary, we firmly believe that droplet microfluidics has evolved into a powerful technology that will pave the way for high-throughput screening of engineered microbial strains in the green biomanufacturing industry.

## Introduction

The continuous increase of global average atmospheric carbon dioxide has led to a serious environmental crisis, and one of the main culprits is the excessive use of fossil fuel energy. Introducing the green biomanufacturing system into traditional chemical processing, with sustainable cell factories for biofuels and commodity chemical production, has fundamentally minimized or prevented toxic pollutants and greenhouse gas emissions (Orsi et al., 2021; Yang et al., 2021). In green biomanufacturing, one pivotal link is the transformation of engineered microbial cells. Its characteristic research application involves genetic modification of microbial chassis, imparting targeted traits and functions for effective synthesis of the desired products (Guo et al., 2022). Furthermore, the rapid acquisition of target strains requires the establishment of a high-throughput screening (HTS) strategy aimed at identifying certain phenotypes such as enzyme activity and specific products yielding (Yuan et al., 2022). Currently, the most widely used screening methods employ compartmentalizing clonal populations in microtiter plate (MTP) wells, which are detected by a microplate plate reader (Böttcher et al., 2017). However, these methods commonly demand sophisticated automated instruments and time-consuming operation procedures, which limit the screening types and efficiency. Fluorescence-activated cell sorting (FACS) allows specific cell populations to be isolated from each mixture with the aid of flow cytometry. The sorting depends on cells’ light-scattering or fluorescent properties, offering a throughput of up to 10^8^ events per hour, as shown in Table 1 (Körfer et al., 2016). Standard FACS can merely capture fluorescent signals from the surface of cell membranes or intracellular products, while not from extracellular secretions.

**TABLE 1:** Differences of MTP, FACS, and DMF for strain screening.

Method	MTP	FACS	DMF
Detection signal	Fluorescence and absorbance	Fluorescence	Fluorescence, Raman, absorbance, and mass spectrometry*.*
Sensitivity	Normal	High	High
Throughput	10^6^/day	10^8^/h	10^8^/day
References	Utharala et al. (2022)	Körfer et al. (2016)	Wang et al. (2021)

**FIGURE 1:**
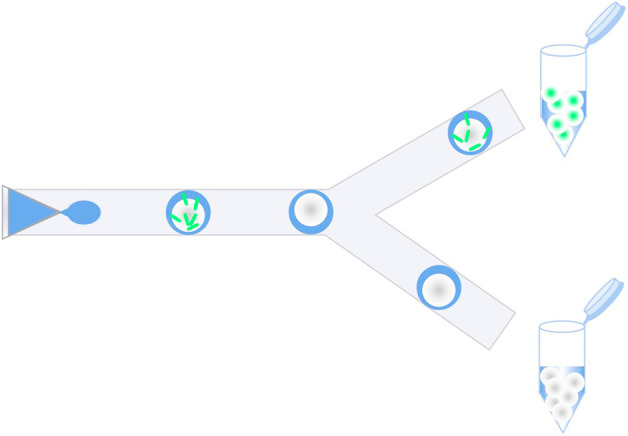
Schematic of high-throughput screening of microbial strains in microfluidic droplets, mainly including mutant library encapsulation and droplet sorting.

Microfluidics, as an emerging complementary solution, focuses on controlling and manipulating fluid in channels at the microscopic scale. One of its subcategories is droplet-based microfluidics (DMF), which generates discrete droplets using immiscible multiphase fluids at kHz frequencies (Wang et al., 2021; Utharala et al., 2022). Each droplet is equivalent to a micro-reactor with a single strain encapsulated inside, thereby facilitating distinct microbial analysis without cross-contamination, as shown in Figure 1. The environment within a single droplet is similar to that of the conventional liquid medium, with sufficient oxygen supply for the growth of individual strains. DMF has revolutionized strain screening due to its ability to precisely handle fluids at length scales comparable to cells (Yuan et al., 2022). For example, a droplet-based microfluidic platform was constructed to rapidly characterize a series of native and heterologous constitutive promoters in *Streptomyces lividans 66* in droplets, and then a set of engineered promoter variants with desired strengths were efficiently screened out from two synthetic promoter libraries (Tu et al., 2021). Before sorting, strains should first be enclosed in droplets, i.e., single-cell encapsulation, kept alive for a certain period of time, and enabled by diversified operations comprising reagent injection, coalescence, lysis, splitting, sorting, *etc.* By this means, the microfluidic droplets possess a uniform and tunable size and a volume ranging from femtoliter to nanoliter, which greatly reduces reagent consumption and the corresponding cost. Therefore, it has been confirmed that droplet microfluidics provides new insights and solutions into practical application in engineered strain modification and mutant host screening. This perspective focuses on the applications of DMF in high-throughput screening of a variety of microbes, including bacteria, yeast, and filamentous fungi; this study discusses the advantages brought by large-scale droplets and presents the future trends.

### Mutant generation and droplet encapsulation

The primary step for microbial strain HTS is constructing large-scale mutant libraries. The more variants available for screening, the greater the possibility of identifying extremely rare but beneficial mutations. Thus, microbial transformation usually utilizes random mutagenesis to accumulate variations with sufficient throughput, involving physical means such as ultraviolet irradiation, atmospheric and room temperature plasma (ARTP), heavy ion radiation, and other chemical mutagenesis approaches (Zeng et al., 2020). Subsequently, the mutant cells are then enclosed in individual droplets, and normal growth is maintained. Most mainstream research studies used the single emulsion system which is based on passive hydrodynamic pressure, and whose microchannel junctions can be classified into flow-focusing, cross-flow, and co-flow configurations (Wang et al., 2022). Concretely, a microbial culture medium with/without the substrate for further detection reactions contains common component similar to the dispersed aqueous phase. Then, the fluids are infused into the continuous oil phase, with favorable biocompatibility and water immiscibility, and break up into monodisperse water-in-oil (W/O) droplets when meeting at the junction. Surfactant addition to the two phases can stabilize the droplets and prevent coalescence. It is worth mentioning that the number of strains in a single droplet is not exactly one, which does not follow the Poisson distribution. High dilution can prevent the internal number from exceeding one, to a certain extent. The size of droplets can be altered by adjusting the channel geometry and the relative flow rates of the two phases. Picoliter droplets are well-suited for loading individual bacterium (∼10 pL), yeast cells (300 pL), characterizing the production or consumption of their extracellular metabolites, and screening monoclonal antibodies produced by hybridoma cells (660 pL) (El Debs et al., 2012; Wang et al., 2014). For large-sized filamentous fungi with well-developed hyphae, nanoliter-level droplets (∼10 nL) are selected for encapsulation, where the strain can keep growing until droplet rupture occurs, pierced by the tip of germinated mycelium (Romero et al., 2015). The droplets’ stability remarkably affects the long-term cultivation of internal strains and subsequent product detection. Nevertheless, the favorable air permeability of the typically used material for microfluidic chips, poly (dimethylsiloxane) (PDMS), can easily cause the volatilization of inner oil fluids and the destruction of the droplet state. Moreover, considering that an *in situ* culture is inconvenient for later detection and sorting, droplets are commonly transferred to centrifuge tubes or syringes for off-line culture.

### Signal screening

Additionally, appropriate screening signals and reaction strategies need to be determined. Since the droplets are transparent, signals of either intracellular or extracellular products can be accurately detected without interference. Ordinary screening signals include fluorescence, absorbance, Raman spectrum, and mass spectrometry (MS), expanding the variety of strains (Markel et al., 2020). Their sources differentiate related droplet operation, including self-products, biosensors, and exogenously added reagents. The products of some microorganisms possess autofluorescence (e.g., carotenoids and riboflavin), so the absorbance and fluorescence intensity of droplets together with internal strains can be directly captured, free from additional reagents and complicated manipulation. Among several signals, the rate of fluorescence-based sorting can reach 300 droplets per second. Also, the Raman and mass spectra of the products can be measured. However, the signal intensity of the former is weak, with a long acquisition time, and the latter needs matching MS analysis, causing a low sorting throughput of approximately 200 per minute (Stucki et al., 2021). When the products lack obvious detectable characteristics, four ingredients can be integrated and co-encapsulated with strains, including fluorescent substrates for enzyme activity determination, fluorescent protein labeling, fluorescent probe coupling, and biosensors enabling sensing target products (Bouzetos et al., 2022). The principal action lies in converting the biological activity into a detectable fluorescent signal. The latest studies emphasize designing a living biosensor. Specifically, the production strain and the sensing strain, the latter of which can homogeneously respond to the former’s products, are co-embedded in the droplet, and then the fluorescent signal emitted by the sensing strain can indirectly reflect the targeted content (Lim et al., 2018; Hua et al., 2022). This approach is appealing for a wide range of unexpected or difficult to genetically manipulate engineered strains.

### Droplets sorting

For cases where the reaction between the substrate reagents and products occurs quickly, the strains are required to first be encapsulated in the droplet and grow for a while for metabolite secretion. After that, the reactant can be added to the droplet through coalescence or injection, fostering the resulting detection and sorting. Droplet fusion can be categorized into passive and active ways. In passive coalescence, precise control over the velocity of individual droplets is a prerequisite to help the droplets gradually approach each other and finally merge; this is achieved by designing the channel geometry. Passive methods are inefficient and error-prone, so active strategies that provide external energy input to apply local actuation, such as electrical, magnetic, thermal, and mechanical forces, are often employed in practical applications. Among them, implementing electric control to induce droplet interface destabilization to aid coalescence or injection is most common, with a throughput of thousands of droplets per second. Similarly, droplets can also be sorted passively based on physical properties, such as size, or actively by on-demand activation. For instance, under dielectrophoretic forces, droplets with strong fluorescent signals are dragged into the downstream collection channel, and the remaining droplets are distributed to the waste channel (Shang et al., 2017).

## Perspective

To date, droplet microfluidics has been successfully applied to a variety of microbes, including bacteria, yeast, and filamentous fungi, and the detection of massive metabolites of strain products, such as polypeptides, enzymes, and lipids, has been realized. By virtue of droplet-based HTS, high-yielding mutants have been obtained. In addition to the expansion of microbial strains, screening signals, and reaction strategies, some innovative ideas emerged, optimized from the perspectives of chip construction, droplet conversion into hydrogel microbeads (Li et al., 2018), and the combination with double emulsion systems (Körfer et al., 2022) and FACS (Li et al., 2021). Despite the promising potential of droplet microfluidics in green biomanufacturing, there are still urgent problems to be solved. Fluorescence is currently the most dominant screening signal, but there still exists a large number of compounds that cannot be detected by fluorescence, so it is necessary to develop other high-efficiency screening signals. In addition, specific strains do not appear to be the smoothly spherical or ellipsoidal but harbor dense mycelial network, such as *Aspergillus niger*, *Streptomyces*, and *Gibberella Fujikura* (Beneyton et al., 2016; He et al., 2019). However, compared to other strains, there are two limiting factors that make the use of droplet microfluidics for filamentous fungi challenging. First, their hyphae tip can readily puncture the droplet and cause leakage of extracellular products. Furthermore, the long droplet incubation times needed for protein expression and the different individual growth rates contribute to droplet volume polydispersity post-incubation, which complicates the sorting procedure. Last but not least, droplets containing mycelium are more susceptible to high-voltage electric fields, which deform and break the droplets (Samlali et al., 2022). Therefore, diverse solutions for optimal droplet morphology and material systems need to be developed to match categories of strains. Moreover, versatile automatic devices integrating functional modules of droplet generation, cultivation, coalescence, reagent injection, and sorting are still lacking, which limits industrial upscaling. In summary, we firmly believe that droplet microfluidics has evolved into a powerful technology that will pave the way for HTS of engineered microbial strains in the green biomanufacturing industry.
